# Partnering With Massage Therapists to Communicate Information on Reducing the Risk of Skin Cancer Among Clients: Longitudinal Study

**DOI:** 10.2196/21309

**Published:** 2020-11-02

**Authors:** Lois Loescher, Kelly Heslin, Graciela Silva, Myra Muramoto

**Affiliations:** 1 College of Nursing University of Arizona Tucson, AZ United States; 2 Colleges of Medicine and Public Health University of Arizona Tucson, AZ United States

**Keywords:** cancer prevention, skin cancer, e-training, electronic intervention, massage therapists

## Abstract

**Background:**

Skin cancer affects millions of Americans and is an important focus of disease prevention efforts. Partnering with non–health care practitioners such as massage therapists (MTs) can reduce the risk of skin cancer. MTs see clients’ skin on a regular basis, which can allow MTs to initiate “helping conversations” (ie, brief behavioral interventions aimed at reducing the risk of skin cancer).

**Objective:**

The purpose of this study was to evaluate (1) the feasibility of recruiting, enrolling, and retaining Arizona MTs in an online electronic training (e-training) and (2) the preliminary efficacy of e-training on knowledge, attitudes/beliefs, and practice of risk reduction for skin cancer. We explored MTs’ ability to assess suspicious skin lesions.

**Methods:**

We adapted the existing educational content on skin cancer for applicability to MTs and strategies from previous research on helping conversations. We assessed the feasibility of providing such e-training, using Research Electronic Data Capture (REDCap) tools for data capture. We assessed the preliminary efficacy using established self-report surveys at baseline, immediately post training, and at 3 and 6 months post training.

**Results:**

A total of 95 participants enrolled in the study, of which 77% (73/95) completed the assessments at 6 months (overall attrition=23%). Project satisfaction and e-training acceptability were high. Knowledge, personal behaviors (skin self-examination, clinical skin examination, sun protection frequency), and practice attitudes (appropriateness and comfort with client-focused communication) of risk reduction for skin cancer improved significantly and were sustained throughout the study.

**Conclusions:**

The e-training was feasible and could be delivered online successfully to MTs. Participants were highly satisfied with and accepting of the e-training. As such, e-training has potential as an intervention in larger trials with MTs for reducing the risk of skin cancer.

**International Registered Report Identifier (IRRID):**

RR2-10.2196/13480

## Introduction

Skin cancer, the most common cancer in the United States, poses a serious public health burden. Over 5.4 million nonmelanoma skin cancers are treated annually [1]. The incidence of melanoma, the most fatal form of skin cancer, increases by about 3% each year among persons aged >50 years [2]. Skin cancer costs exceed billions of dollars annually [3]. Fortunately, the skin cancer burden can be abated through primary prevention and early detection.

Protecting the skin from ultraviolet radiation reduces the risk of skin cancer [4,5]. Recommended primary prevention behaviors are as follows: avoid prolonged sun exposure during peak intensity, cover the skin with tightly woven protective clothing (eg, long sleeves/pants, wide-brimmed hats, and sunglasses), seek shade, apply/reapply sunscreen, and avoid all kinds of intentional tanning [6]. Early skin cancer detection decreases potential morbidity, mortality, and cost [2,3] and can be carried out with viewable skin assessment (VSA) by health professionals or with skin self-examination (SSE) by consumers. Skin lesions can be assessed using several approaches, one of which is the common asymmetry, border, color, diameter, evolving (ABCDE) rule [7].

One approach for delivering risk-reducing information on skin cancer is a structured “helping conversation,” a person-centered communication technique that emphasizes on active listening and motivational communication to encourage healthy behavior change [8]. A helping conversation consists of 4 steps: awareness, understanding, helping, and relating [9]. Helping conversations are context-specific and thus cost-effective and time efficient [8]; they have been used for other preventive behaviors [8,10] but not in the context of risk reduction for skin cancer.

The *Surgeon General’s Call to Action to Prevent Skin Cancer* [6] endorses community partnerships for reducing the risk of skin cancer. Community partners who can implement helping conversations about reducing the risk of skin cancer include massage therapists (MTs). Compared to primary care providers or dermatologists, MTs are more likely to have repeated and longer appointments that are oriented toward health promotion [11-13], thereby providing greater opportunities for helping conversations. MTs uniquely access most of a client’s skin, allowing VSA. Approximately 385,000 MTs and MT students nationwide, provided with training, have the potential to engage in helping conversations [14].

Some MTs receive education on skin cancer during primary training (60%) and continuing education (25%) [15]; however, the content, duration, and source of this education vary. The few existing skin cancer–focused in-person workshops and 1 online course do not include training for client-focused communication on reducing the risk of skin cancer [15]. There is a need to assess the feasibility of providing such training to MTs, particularly considering the popular press stories of their involvement in early detection [15-17].

Our goal was to develop and evaluate the feasibility of delivering online e-training on reducing the risk of skin cancer, to MTs within the context of a helping conversation. Specific aims of this study were as follows:

1. Assess e-training feasibility, namely, facilitators and barriers to recruitment and enrollment, intervention completion and acceptability, and client acceptability of helping conversations.
2. Analyze the preliminary efficacy of e-training preliminary efficacy in terms of knowledge, personal/practice-based attitudes/beliefs, and practice factors of risk reduction for skin cancer, from baseline to immediately post training and 3 and 6 months post training.
3. Explore the assessment of suspicious versus nonsuspicious skin lesions.

## Methods

The University of Arizona Institutional Review Board approved this study. Data collection was performed between July 1, 2018, and April 1, 2020. Data analysis was completed on April 27, 2020. The study had 3 phases. In phase 1, we developed e-training content, assessments, and technology. In phase 2, we conducted a feasibility study (recruitment, screening, and enrollment; e-training implementation; and assessment of the main variables at 4 time points). In phase 3, we conducted data analysis and interpretation. We have previously published the protocol for this study [9], which is briefly summarized below.

### Conceptual Framework

Social cognitive theory (SCT) posits that individuals learn and maintain new behaviors through reciprocal interaction of person, environment, and behavior [18]. This study was guided by 4 SCT constructs: (1) reciprocal interaction of MTs, their external social contexts, and behavioral responses to the e-training; (2) behavioral capability to have a helping conversation; (3) observational learning from e-training vignettes; and (4) self-efficacy for mastery of knowledge and practice changes.

### Study Population

We initially recruited a single cohort of MTs through professional networking, social media posts (on Facebook), flyer postings at MT practices, peer referral, massage school listservs, and online newsletter postings. Eligibility criteria were as follows: age ≥21 years, licensed in Arizona, practicing for at least 3 years, averaging at least 5 clients per week, and internet access. Eligible MTs provided informed consent prior to enrollment. Participants received US $200 for the 6 months of participation and continuing education credit units.

### Sample Size

Sample size estimations were based on prior studies of skin cancer training for medical students [19] and our research on helping conversations about tobacco cessation with MTs [10]. A repeated measures power analysis for proportions (effect size 16% at pretest and 51% at posttest) indicated that 40 MTs would be needed (α=.05; β=.9). The reported attrition from online trainings ranged from 20% to 80% [20,21]. Enrolling 80 MTs would allow for attrition and reasonable estimation of sample size and recruitment and attrition in a future trial [19]. Power analyses were conducted using PASS software (version 12) [22].

### Intervention

The e-training was built on previously developed skin cancer prevention e-training [23] and established competencies of helping conversations [8]. Participants sequentially completed 6 asynchronous, self-paced modules: introduction, awareness, understanding, helping, relating, and closing. After the modules, they completed 5 simulations of MT-client encounters, reflecting helping conversations that could occur during a 60-minute, full-body massage.

### Measures

Using Research Electronic Data Capture (REDCap) tools hosted at the University of Arizona [24,25], we collected data at 4 time points: baseline (at enrollment), 1 week post training, 3 months post training, and 6 months post training. REDCap is a secure, web-based software platform for supporting data capture for research studies, providing (1) an intuitive interface for validated data capture, (2) audit trails for tracking data manipulation and exporting, (3) automated export procedures for seamless data download to common statistical packages, and (4) procedures for data integration and interoperability with external sources.

To assess e-training feasibility, we maintained detailed recruitment and enrollment records, coding how each potential participant learned of the study. This allowed us to determine the participant yield from each strategy. We used REDCap reporting capabilities to assess e-training completion rates.

Participants responded to 8 e-training satisfaction items (5-point scale, completely unsatisfied=1 to completely satisfied=5). To assess acceptability of the helping conversations of participating MTs, clients completed a 10-item anonymous survey accessible via a quick response (QR) code embedded in the flyers posted at the participating MTs’ practices. If a helping conversation occurred, then the client selected topics mentioned by the MT, along with their recommendations (5-point scale, strongly disagree=1 to strongly agree=5). Clients received a US $5 gift card for participating.

To assess the preliminary efficacy of e-training, we measured general knowledge on skin cancer with 16 multiple-choice items adapted from previous research (scored as correct or incorrect) [26] and 1 item measuring knowledge of the ABCDE rule (scored as correct or incorrect). We measured self-efficacy using a 10-item general self-efficacy scale [27] (4-point scale, not at all true=1 to exactly true=4; total score is calculated by finding the sum of all items and ranges between 10 and 40 with a higher score indicating more self-efficacy). Personal beliefs and attitudes were measured with 1 item for assessing participants’ perceived probability of getting skin cancer in the future (scored from 0%-100%), 5 items for appropriateness of including information about reducing the risk of skin cancer in client interactions, and 3 items about their own comfort with and confidence during those interactions (5-point scale, strongly disagree=1 to strongly agree=5).

We measured participants’ personal behaviors of skin cancer risk reduction with 5 items pertaining to the frequency of sun protection behaviors (5-point scale, never=1 to always=5) and 1 item each for tanning booth visitation (5-point scale, in the past month=1 to never=5), SSE (5-point scale, never=1 to more than once a month=5), VSA (5-point scale, never=1 to more than once a month=5), and clinical skin examination (5-point scale, never=1 to more than once monthly=5).

Using a 29-item questionnaire on case-based image assessment adapted from a medical continuing education training [28], we measured participants’ ability to assess skin lesions. Participants viewed photos of skin lesions accompanied by brief case descriptions, scoring each image as suspicious or not suspicious (scored as correct or incorrect).

We invited participants based in Tucson, Arizona, (n=10) to an in-person posttraining debriefing. We asked them about their overall e-training experience (key takeaways, application in practice, and confidence regarding helping conversations). We asked whether they had noticed any suspicious lesions on a client’s skin during study participation and how we could improve the e-training. Study personnel took detailed notes and compiled the comments.

### Statistical Analysis

We assessed all data for missing and outlier values, deleting missing values listwise and double-checking and verifying outlier data. To describe demographic variables, we computed frequencies and means. We also computed individual composite scores, which summed the average answer value for each item of each of the assessment questionnaires: self-efficacy, personal beliefs and attitudes, and frequency of sun protection behavior.

We scored general skin cancer knowledge questions as percentages of correct or incorrect answers and computed mean scores for questions with continuous answers, such as perceived probability of getting skin cancer. We computed the percentages of correct and incorrect answers for image assessments. Using repeated measures analysis of variance, we evaluated longitudinal differences in composite scores for each of the measures; percentage of correct answers; and continuous variables at baseline, immediately post training, and at 3 and 6 months post training. We used Intercooled Stata, version 15 (Stata Corp), and applied a significance level of .05 for all statistical tests.

## Results

A total of 95 participants enrolled in the study: 77% (73/95) completed all assessments at 6 months. The final sample had a mean age of 46 years; was predominantly female (93%), non-Hispanic or Latino (89%), and White (83%); worked part-time; and saw <11 new or returning clients per week (Table 1). There were no major differences in the demographic characteristics between participants who completed the e-training and those who completed the training and all assessments.

**Table 1:** Demographic characteristics of the sample (N=73).

Characteristics		Values	
Age in years, mean (SD)		46 (12)	
Hours worked per week, mean (SD)		23 (10)	
Number of new clients per week, mean (SD)		9 (9)	
Number of returning clients per week, mean (SD)		11 (7)	
**Gender, n (%)**			
	Male		5 (7)
	Female		68 (93)
**Ethnicity, n (%)**			
	Hispanic or Latino		8 (11)
	Non-Hispanic or Latino		64 (89)
	Prefer not to answer		0 (0)
**Race, n (%)**			
	American Indian or Alaskan Native		4 (6)
	Asian		3 (4)
	Black or African American		4 (6)
	Native Hawaiian or other Pacific Island		1 (1)
	White		60 (83)
	Prefer not to answer		0 (0)
Personal history of skin cancer = yes, n (%)		7 (9)	
Family history of skin cancer = yes, n (%)		40 (55)	

**Figure 1:**
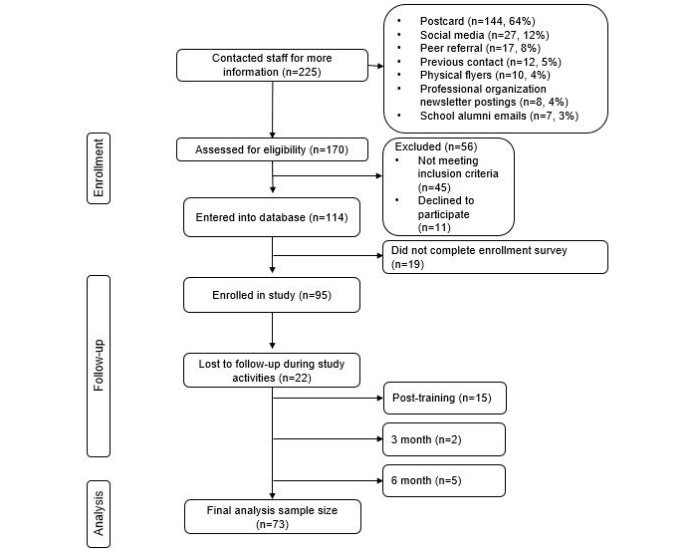
CONSORT (Consolidated Standards of Reporting Trials) flow diagram.

During recruitment, 225 MTs requested study information. Out those, 170 (68%) underwent eligibility screening, and 95 (42%) were enrolled (see Figure 1 for recruitment yield). Overall attrition following enrollment was 23% (Figure 1). Participant acceptability of the e-training and study procedures are shown in Table 2.

A total of 57 clients reported visits with 9 participating MTs (who did not vary in demographics from the overall sample). Clients reported 55 helping conversations, primarily focusing on skin cancer prevention. Conversations mentioned the topics of sunscreen (91%), protective clothing (76%), and wide-brimmed hats (74%). Clients agreed that MTs appropriately initiated the helping conversation (mean 4.41, SD 0.92). Clients were accepting of questions by their MT about sun safety and sun protection behaviors (mean 4.09, SD 1.35), SSE behaviors (mean 3.78, SD 1.65), suggestions regarding skin cancer prevention (mean 4.35, SD 0.99), and shared information about skin cancer prevention (mean 4.45, SD 0.97). Clients were less accepting of queries about marks on their skin (mean 2.30, SD 2.33) and referral to a dermatologist (mean 2.92, SD 2.26).

**Table 2:** Project satisfaction and attitudes toward training (N=73).

Criteria		Values, mean (SD)
**Project satisfaction (1=completely unsatisfied; 5=completely satisfied) (N=73)**		
	Overall, how satisfied were you with participation in this project?	4.4886 (0.99)
	How would you rate the quality of the information you received as part of this project?	4.4953 (0.62)
	How would you rate the value of this project based on the amount of time you participated?	4.5022 (0.84)
	How responsive were study staff to your questions or concerns about the project?	4.5092 (0.69)
	How likely are you to continue using the skin cancer risk reduction knowledge you learned?	4.5024 (0.52)
	How likely are you to continue to use the helping conversation skills you learned?	4.4955 (0.67)
	How likely is it that you would recommend this training to a colleague?	4.5024 (0.90)
	Overall, how satisfied were you with the training?	4.47 (0.65)
	Scale mean	4.51 (0.01)
**Attitudes toward training (1=strongly disagree; 5=strongly agree) (N=81)**		
	I would take this training only if continuing education units were offered.	2.7 (1.12)
	The time commitment required for the training was realistic.	4.31 (0.75)
	The training met my personal expectations.	4.12 (0.84)
	I would take this training without receiving an incentive.	3.73 (0.99)
	I learned more than I knew before about skin cancer risk reduction.	4.14 (0.96)
	I trusted the information I received from the training.	4.49 (0.74)
	The content of the training was useful for my daily life.	4.43 (0.78)
	The training helped build my self-confidence.	3.9 (0.96)
	I would recommend this training to others.	4.38 (0.81)

During the debriefing session, participants stated that the e-training component of the helping conversation was the easiest to remember. Helping conversations were new knowledge to most participants, encouraging communication skills that were not emphasized during their MT education. A common theme was posttraining confidence in discussing skin cancer risk reduction. Participants also found that their existing clients were more accepting of conversations than new clients. The most common conversation topics were prevention-oriented, such as reducing sun exposure; these conversations often started with variations of the example phrases provided in the e-training (eg, “what do you do to protect yourself from the sun?”). Moreover, 2 MTs reported mentioning suspicious lesions to clients, which were later diagnosed as skin cancers by a dermatologist.

Preliminary efficacy results for knowledge, personal and practice beliefs, and behaviors are shown in Table 3 along with the exploratory findings for the case-based image assessment. During the in-person debriefing session, participants commented that they would have preferred more examples of suspicious skin lesions and correct answers to each case. Participants felt there was no harm in labeling every image as suspicious or eliciting concern to avoid missing any important findings.

**Table 3:** Knowledge, personal beliefs/attitudes, and practice attitudes.

Criteria		Composite scores of correct answers				*P* value^a^	
		Baseline	Posttest 1	Posttest 2	Posttest 3		
**Knowledge**							
	General skin cancer knowledge	0.57	0.74	0.71	0.71		<.001
	ABCDE^b^ knowledge	0.64	0.68	0.68	0.83		.002
	Case-based image assessment	0.74	0.72	0.72	0.72		.04
**Personal beliefs/attitudes**							
	Prevention beliefs	3.79	3.92	3.63	3.59		<.001
	Prevention behavior: SSE^c^	2.97	3.23	3.42	3.53		<.001
	Prevention behaviors: clinical skin examination	1.99	2.08	2.27	2.33		<.001
	Frequency of sun protection behavior	3.73	3.82	3.84	3.84		.03
	General self-efficacy	34.54	34.08	33.95	34.45		.19
	Perceived potential risk of skin cancer (on the scale of 1 to 100)	44.54	43.29	39.46	40.91		.10
**Practice attitudes**							
	Appropriateness of client interactions	3.97	4.33	4.22	4.33		<.001
	Confidence in client interactions	4.11	4.48	4.44	4.46		<.001
	Confidence in protecting one’s own skin	3.43	3.52	3.53	3.55		.18
	Confidence in assessing one’s own skin	3.34	3.5	3.48	3.51		.08

^a^Statistical significance (*P*<.05) for repeated measures analysis of variance.

^b^ABCDE: asymmetry, border, color, diameter, evolving.

^c^SSE: skin self-examination.

## Discussion

### Feasibility

The key finding was that the e-training was feasible and could be delivered online successfully. Similar to our pilot study [9], participants were highly satisfied with and accepting of the e-training. Clients were also satisfied with their MTs discussing skin cancer–prevention topics.

The main challenge was recruitment. Previous studies of MTs recruited participants attending massage therapy conferences [15,29]. To the best of our knowledge, this study was the first to recruit MTs for research from statewide independent, group, and national chain practices. Our initial multipronged recruitment efforts resulted in a surge of interested MTs that lasted 2 months. Contacts then lagged until we contacted the Arizona Chapter of the American Massage Therapy Association, purchased their mailing list, and mailed recruitment postcards to members as a secondary strategy. Contrary to previously reported observation of higher success with using social media versus direct mail to recruit hard-to-reach populations [30], the mailed postcard was the most effective recruitment strategy in our study. We did not ask participants why this strategy was successful but surmised that they preferred a direct outreach approach with targeted mailings [31] or did not tend to engage in social media.

Despite recruitment challenges, participants tended to stay in the study. According to the debriefing comments, once they completed the e-training, incentives such as continuing education credit and their own desire to add to the body of knowledge of their profession maintained their participation. This thinking reflected surveyed attitudes toward the e-training, with participants indicating that they would take the training without incentives and continuing education credit. This form of altruism is not unusual in community-based research, where participants desire to have a connection to science and their profession [32]. However, we would recommend incentives for future studies of this scope.

Collecting client survey responses was another challenge. MT participants received flyers that provided access to the client survey for posting in their practices; however, we had limited knowledge of whether they posted those flyers. We concur with others that flyers have limited utility [33]. At the 3- and 6-month evaluations, we asked MTs to remind clients about the survey, which appeared to improve the client survey response.

### Preliminary Efficacy

The key finding was that participants’ knowledge, personal behaviors (SSE, clinical skin examination, and frequency of sun protection), and practice attitudes (appropriateness and comfort with client-focused communication) of risk reduction for skin cancer improved and were sustained throughout the duration of the study. Despite these positive findings, there are some persistent issues.

Improved skin cancer knowledge is consistent with findings of previous education interventions for skin cancer provided to MTs or cosmetologists [29,34]. Although knowledge improved, it improved to a barely “passing score,” which reflected the passing score (70%) required for the Massage & Bodywork Licensing Examination (MBLEx) [35]. The most common incorrect knowledge answers selected at 6 months were the strongest risk factor for melanoma, indices important for reducing exposure to ultraviolet radiation, and recommended sunscreen ingredients. We are considering adding “boosters” to the revised e-training to further improve knowledge that is critical to helping conversations.

Attitudes favorably increased over the duration of the intervention except for self-efficacy and perceived risk of skin cancer. The general self-efficacy scale reflects a belief that an individual is capable of performing novel or difficult tasks and coping with adversity [27]. Self-efficacy scores did not significantly change over the 3 data collection points. Although participants had positive general self-efficacy, they were neutral in their confidence for protecting and assessing their own skin. They were more comfortable discussing skin cancer risk reduction with their clients, which reflects findings from other studies [15]. A situation-specific self-efficacy measure may be more useful in characterizing MTs’ self-confidence, particularly that of helping conversations. A perceived risk of skin cancer is most commonly considered a stable belief; however, it could change under specific circumstances, such as a personal skin cancer diagnosis [36].

### Exploratory Aim: Case-Based Image Assessment

We explored whether participants could assess a suspicious versus nonsuspicious skin lesion using the ABCDE rule. Although participants’ understanding of the rule improved over time, mean correct scores on the case-based image assessment at all time points fell in the range of 72%-74%. Trotter et al [29] had a similar finding in their image assessment using just 4 images. Participants in our study tended to correctly score “ugly duckling” (unsightly) lesions and incorrectly score nonsuspicious lesions. This pattern has been reported in other studies of case-based image assessments [29]. The drop in scores following the training may be attributable to MTs’ opinions mentioned during the debriefing session, where they would “rather be safe than sorry” and were likely to select all images as suspicious and warranting referral. Throughout the study, MTs shared their desire for more images of skin cancers as well as the correct answers to the image assessment. Although the latter was not feasible as per the study design, future e-training content will use a larger image bank that will enable us to alternate image choices during data collection and provide immediate feedback.

### Additional Limitations

There is some missing data in our analysis due to our failure to initially force item responses and initially develop asynchronous/chained survey invitations when designing the online instruments. We added questions (MT practice type and asking for more specific training feedback) shortly after the enrollment began, further resulting in missing data from the previous version. In total, 3 participants failed to fully complete a total of 7 surveys between them. Our Arizona-specific sample also limits generalizability to MTs in other geographic areas. The heterogeneity of MT practice models (eg, sole proprietor, employee, partner, and independent contractor) made it challenging to recruit participants and collect data for client surveys. Noninclusion of a control group limits the strength of our findings and will be incorporated in future research.

### Conclusions

Our results demonstrated that it is possible to engage practicing MTs in a skin cancer education study and that MTs will complete e-training. We also demonstrated that although MTs completed 2-hour-long, case-based e-training, it was not sufficient to significantly increase their self-efficacy in initiating helping conversations about skin cancer with clients or their ability to recognize images of skin cancers. The e-training increased their knowledge about skin cancer prevention and early detection.

## Abbreviations

ABCDE: asymmetry, border, color, diameter, evolving
e-training: electronic training
MBLEx: Massage & Bodywork Licensing Examination
MT: massaging therapist
QR: quick response
SCT: social cognitive theory
SSE: skin self-examination
VSA: viewable skin assessment
